# Rapid and Ultrasensitive Quantification of Multiplex Respiratory Tract Infection Pathogen via Lateral Flow Microarray based on SERS Nanotags

**DOI:** 10.7150/thno.35824

**Published:** 2019-07-09

**Authors:** Di Zhang, Li Huang, Bing Liu, Qinyu Ge, Jian Dong, Xiangwei Zhao

**Affiliations:** 1 State Key Laboratory of Bioelectronics, School of Biological Science and Medical Engineering, Southeast University, Nanjing 210096, China; 2 National Demonstration Center for Experimental Biomedical Engineering Education, Southeast University, Nanjing 210096, China; 3 Department of Biomedical Engineering, Yale University, New Haven, CT 06520, USA; 4 Getein Biotechnology Co., Ltd., Nanjing 210000, China

**Keywords:** lateral flow microarray, core-shell SERS nanotags, multiplex nucleic acids detection, respiratory tract infection (RTI)

## Abstract

Respiratory tract infections (RTIs) are severe acute infectious diseases, which require the timely and accurate identification of the pathogens involved so that the individual treatment plan can be selected, including optimized use of antibiotics. However, high throughput and ultrasensitive quantification of multiple nucleic acids is a challenge in a point of care testing (POCT) device.

**Methods:** Herein, we developed a 2×3 microarray on a lateral flow strip with surface enhanced Raman scattering (SERS) nanotags encoding the nucleic acids of 11 common RTI pathogens. On account of the signal magnification of encoded SERS nanotags in addition to the high surface area to volume ratio of the nitrocellulose (NC) membrane, rapid quantification of the 11 pathogens with a broad linear dynamic range (LDR) and ultra-high sensitivity was achieved on one lateral flow microarray.

**Results:** The limit of detection (LOD) for influenza A, parainfluenza 1, parainfluenza 3, respiratory syncytial virus, coxiella burnetii, legionella pneumophila, influenza B, parainfluenza 2, adenovirus, chlamydophila pneumoniae, and mycoplasma pneumoniae were calculated to be 0.031 pM, 0.030 pM, 0.038 pM, 0.038 pM, 0.040 pM, 0.039 pM, 0.035 pM, 0.032 pM, 0.040 pM, 0.039 pM, and 0.041 pM, respectively. The LDR of measurement of the target nucleic acids of the eleven RTI pathogens were 1 pM-50 nM, which span 5 orders of magnitude.

**Conclusions:** We anticipate this novel approach could be widely adopted in the early and precise diagnosis of RTI and other diseases.

## Introduction

Respiratory tract infections (RTIs), which include acute upper respiratory tract infection, acute tracheitis, acute bronchitis, bronchiectasis, and pneumoniae, are severe acute infectious diseases. Any time delay in RTI diagnosis will exacerbate the patient's condition and can even lead to their death. In addition, inaccurate diagnosis can lead to the over-prescribing of antibiotics. Therefore, timely and accurate detection of the pathogen infecting a patient not only forms the basis of diagnosis but is also fundamental to the selection of an individual treatment plan and standardized use of antibiotics. However, it is normally difficult to undertake an accurate clinical diagnosis for RTIs because many different pathogens can be involved, including bacteria and atypical pathogens. A single clinical manifestation can be caused by many different pathogens and conversely, one pathogen can give rise to a variety of clinical manifestations. It has been documented that 80% of upper respiratory tract infections (URTIs) and a proportion of lower respiratory tract infections (LRTIs) are caused by atypical pathogens 1, 2, including influenza A, influenza B, parainfluenza 1, parainfluenza 2, parainfluenza 3, adenovirus, respiratory syncytial virus, chlamydophila pneumoniae, coxiella burnetii, mycoplasma pneumoniae, legionella pneumophila, *etc*. These pathogens often co-exist when RTI occurs. Traditional methods for confirming the atypical pathogens are mostly dependent on laboratory examination following pathogen isolation and culture, which is complicated, time-consuming, difficult, and with a low limit of detection (LOD). Currently, such methods are being replaced by rapid diagnostic techniques using immunological biomarkers. For example, a commercial point of care testing (POCT) product (Vircell, Spain) can detect the nine atypical pathogens described above using an indirect immunofluorescence assay (IFA), based on the detection of IgM antibodies in human serum. However, there is a time period during which IgM antibodies cannot be detected after infection by the pathogens in humans, in addition to the level of antibody production being dependent on an individual's level of immunity. Thus, a negative result cannot rule out infection by a particular RTI pathogen. Furthermore, the sera of patients with autoimmune diseases may trigger a nonspecific cellular reaction using the IFA method, resulting in false positive results. Hence, diagnosis using a commercial POCT product must be confirmed with other methods which does not fundamentally solve the problem of rapid and accurate diagnosis of the RTIs. In contrast to antibody detection, the identification of nucleic acids can be relatively accurate because those of an infecting pathogen are distributed around the human body, especially within saliva.

A lateral flow assay (LFA) is among the most extensively utilized form of analysis within POCT devices 3-9. The common method of achieving multiplex detection in an LFA is a parallel test (T) line arrangement on a nitrocellulose (NC) membrane, each line dedicated to the identification of a single biomarker 10, 11. However, the finite dimensions of an LFA restricts the scope of high throughput detection without expansion of the LFA strip. There are a number of studies that describe the construction of a lateral flow microarray (LFM) that combines a microarray with an LFA. This greatly increases detection throughput, with as many as eight biomarkers able to be analyzed per strip 12, 13, although the colloidal gold labels used in these assays restrict the quantitative capability and sensitivity of an LFM. In addition, the size of the strip restricts the quantity of dots for each microarray and it is not possible to greatly increase the throughput of detection of the LFM to any great extent. Therefore, an LFM for RTI-related detection of nucleic acids with ultra-high sensitivity, a short sample-to-result time and enhanced multiplex detection capacity is in high demand.

**Figure 1:**
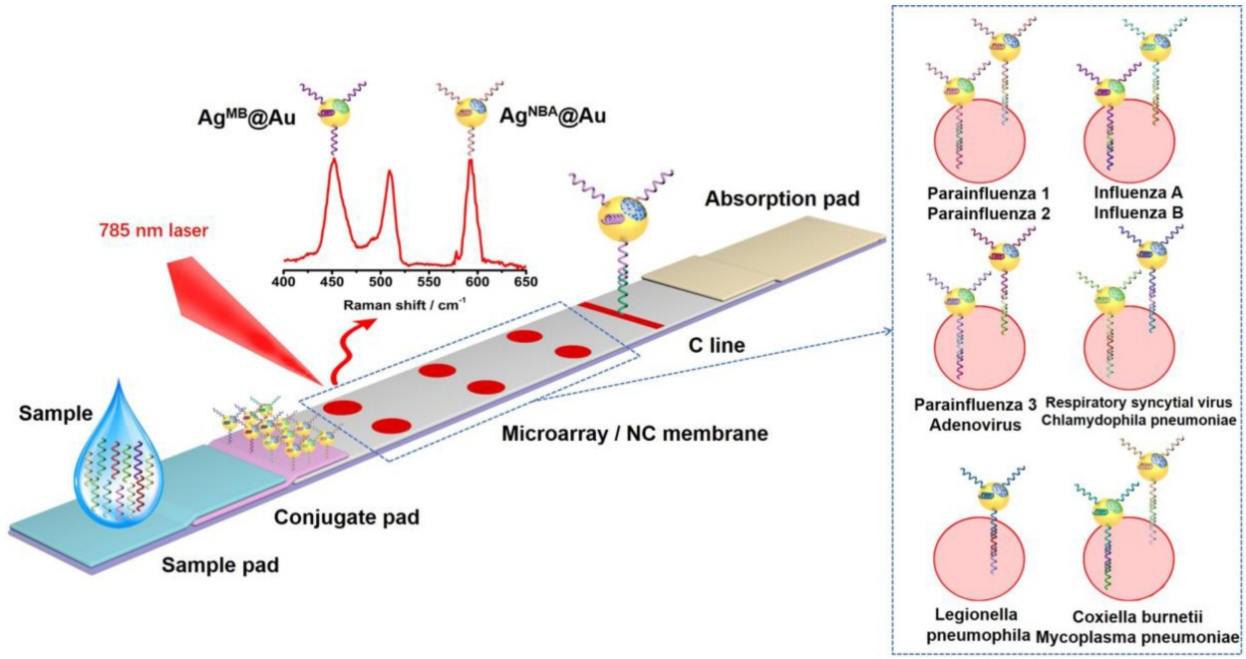
Schematic illustration of SERS LFM for the detection of the nucleic acids of eleven RTI pathogens with RDs encoded core-shell SERS nanotags.

Surface enhanced Raman scattering (SERS) LFA has been demonstrated to be capable of extremely sensitive quantification, according to research performed by our laboratory and others 14-17. Encoded SERS nanotags can be used to accomplish multiplex detection on a single T line in an LFA 18, an arrangement that has been used to detect proteins and nucleic acids 19 with the potential for *in vitro* diagnostic (IVD) detection. Herein, we constructed a SERS LFM strip that had the capability to simultaneously detect the nucleic acids of eleven atypical pathogens (influenza A, influenza B, parainfluenza 1, parainfluenza 2, parainfluenza 3, adenovirus, respiretory syncytial virus, chlamydophila pneumoniae, coxiella burnetii, mycoplasma pneumoniae and legionella pneumophila) commonly found in RTIs. As displayed in Figure 1, silver core gold shell bimetallic nanoparticles with the Raman dyes (RDs) NBA (Nile blue A) and MB (methylene blue) were encapsulated into their interior surface to obtain encoded SERS nanotags (Ag^MB^@Au and Ag^NBA^@Au). Detection nucleic acids of the eleven RTI pathogens were linked with the encoded SERS nanotags. Eleven capture nucleic acids representing the eleven RTI pathogens were incorporated into a 2×3 microarray (six T dots) on the NC membrane with each of the five T dots detecting two nucleic acids and one T dot detecting just one. After sample acquisition has been completed, Raman signals from the T dots can be inspected individually for quick quantification of the eleven pathogen nucleic acids. As far as we know, this is the first time simultaneous quantitative detection of up to eleven pathogenic nucleic acids has been achieved using one SERS LFM strip.

## Material and methods

### Materials and chemicals

HS-PEG-COOH (Thiolated-carboxylated PEG, MW ∼5 kDa) was got from Laysan Bio, Inc. (USA). HAuCl_4_·3H_2_O (Tetrachloroaurate (III) trihydrate), NH_2_OH·HCl (hydroxylamine hydrochloride), AgNO_3_ (silver nitrate), Na_3_C_6_H_5_O_7_ (trisodium citrate), and NaBH_4_ (sodium borohydride) were purchased from Sigma-Aldrich. Methylene Blue (MB), sucrose, tween-20, Nile blue A (NBA), NHS (sulfo-N-hydroxysuccinimide), and EDC (ethyl dimethylaminopropyl carbodiimide) were got from Alfa Aesar. Absorption pad, sample pad, NC membrane, gold conjugate pad, TE (Tris and EDTA) buffer, 0.01 M phosphate buffer (pH 7.0), and streptavidin were provided by GeteinBiotech (China). All the nucleic acid oligonucleotides used in this study were purchased from GenScript (China) and the sequences are shown in Table 1. Blank throat swab samples were gathered from Zhongda Hospital Southeast University, approved by its Institutional Ethics Committee. All water was purified to Milli-Q quality (Millipore, 18 MΩ).

### Preparation of core-shell SERS nanotags

HS-PEG-COOH encapsulated Ag^MB^@Au nanoparticles (NPs) and Ag^NBA^@Au NPs were prepared in accordance with methods previously developed in our laboratory 14, 18. The RDs (NBA and MB) were conjugated to Ag NPs respectively through electrostatic absorption. The gold shell was formed on Ag@NBA and Ag@NBA by seeds-mediated growth method. After the formation of HS-PEG-COOH encapsulated Ag^MB^@Au NPs and Ag^NBA^@Au NPs, 5 μL 110 mg mL^-1^ NHS and 5 μL 40 mg mL^-1^ EDC were mixed with the NPs (Ag^NBA^@Au and Ag^MB^@Au) then stirred vigorously for 20 min at 25°C. After centrifugation, the supernatant was discarded and the NPs were re-suspended in PB buffer to the original volume. The amino groups of the detection nucleic acids were then reacted at 25°C for 2 h with the activated PEG-capped NPs, the nucleic acids of influenza A, parainfluenza 1, parainfluenza 3, respiratory syncytial virus, coxiella burnetii, legionella pneumophila with Ag^NBA^@Au NPs and those of influenza B, parainfluenza 2, adenovirus, chlamydophila pneumoniae, mycoplasma pneumoniae with Ag^MB^@Au NPs. After three rounds of centrifugation, the NPs were retrieved then preserved in TE buffer at 4°C until required for future use.

### Preparation of SERS LFM strips

A schematic diagram of the preparation of SERS LFM strips is shown in Figure 1. A mixture comprising equal molarities of influenza A, parainfluenza 1, parainfluenza 3, respiratory syncytial virus, coxiella burnetiid and legionella pneumophila conjugated Ag^NBA^@Au SERS nanotags and influenza B, parainfluenza 2, adenovirus, chlamydophila pneumoniae, mycoplasma pneumoniae conjugated Ag^MB^@Au SERS nanotags, in addition to control nucleic acid linked with Ag^NBA^@Au NPs were fixed to the conjugate pad. The final concentration of each SERS nanotag was 1.3×10^-6^ M. In order to prepare the test (T) microarrays, equal molar mixture of streptavidin connected biotin decorated influenza A and influenza B, parainfluenza 1 and parainfluenza 2, parainfluenza 3 and adenovirus, respiratory syncytial virus and chlamydophila pneumoniae, coxiella burnetii and mycoplasma pneumoniae as well as legionella pneumophila were spotted on the NC membrane to form a 2×3 microarray through a home-made tapered capillary combined with a micromanipulator system. The complete device is displayed in Figure S1. A complementary chain of control nucleic acid was immobilized onto the NC membrane to form a C (control) line. Finally, the strip was cut to a width of 5 mm until required for testing.

### Analysis of the SERS LFM

To ascertain the principal characteristics of the SERS LFM, samples spiked with different concentrations of various target pathogen nucleic acids were prepared. An aliquot of sample (100 μL) was added to the sample pad. As the sample flowed through the conjugate pad, the target pathogen nucleic acids associated with the complementary nucleic acids fixed to the surface of the SERS nanotags (Ag^NBA^@Au and Ag^MB^@Au), producing eleven diverse forms of complex. As these complexes arrived at the microarrays, the complexes were captured by the capture nucleic acids that were pre-immobilized on the various microarray cells, forming a sandwich hybridization complex. Aggregation of the SERS nanotags (Ag^NBA^@Au NPs and Ag^MB^@Au NPs) resulted in a distinctive red dot on the NC membrane. Remaining solution continued to flow along the strip, with control nucleic acid-linked Ag^NBA^@Au NPs becoming trapped by the complementary control nucleic acid via the specific combination on the C line. After 7 minutes, a color change of the SERS nanotag labels on the C line, in addition to the test microarray labels could be observed by naked eye. A clear red band appearing on the C line established that the SERS LFM strip was operating correctly. The presence of nucleic acids from one of the pathogens could be verified not only from the intensity of the SERS signal but also by the change in color of the associated microarray T dot. Quantification of pathogenic nucleic acid was accomplished by measuring the intensity of the SERS signals of the Ag^MB^@Au NPs and Ag^NBA^@Au NPs on the T dots.

## Results and Discussion

### Synthesis of silver-gold core-shell SERS nanotags

**Figure 2:**
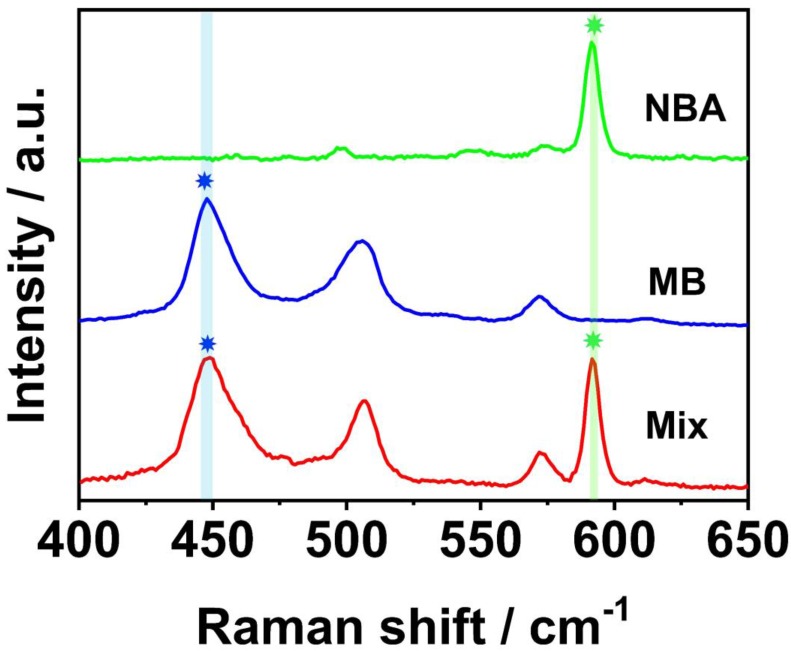
Raman spectra of the Ag^NBA^@Au (green line) and Ag^MB^@Au (blue line) NPs, and an equimolar mixture (red line). The highlighted Raman peaks at 448 and 592 cm^-1^ are unique to MB and NBA, respectively.

In the SERS LFM described here, diverse RDs encapsulated in silver core gold shell SERS nanoparticles acted as encoded labels for ultrasensitive and multiplex quantification of nucleic acids. Hence, the coding capacity and corresponding Raman signal intensities of the SERS nanoparticles should be fully considered. Here, Nile blue A (NBA) and methylene blue (MB) RDs were selected for the reasons described in previous studies 18, 20. The Raman spectra derived from the Ag^NBA^@Au, Ag^MB^@Au and equimolar mixtures of Ag^MB^@Au and Ag^NBA^@Au NPs are shown in Figure 2. Characteristic SERS peaks of MB (448 cm^-1^) and NBA (592 cm^-1^) demonstrated that no overlap in signal occurred and that the Raman intensity was similar, allowing the raw SERS spectra of the mixture to be quantified without additional spectrum deconvolution 21, 22. Based on the above evaluation, Ag^MB^@Au and Ag^NBA^@Au NPs were selected for use in the SERS LFM.

### Size and signal uniformity of the microarray

**Figure 3:**
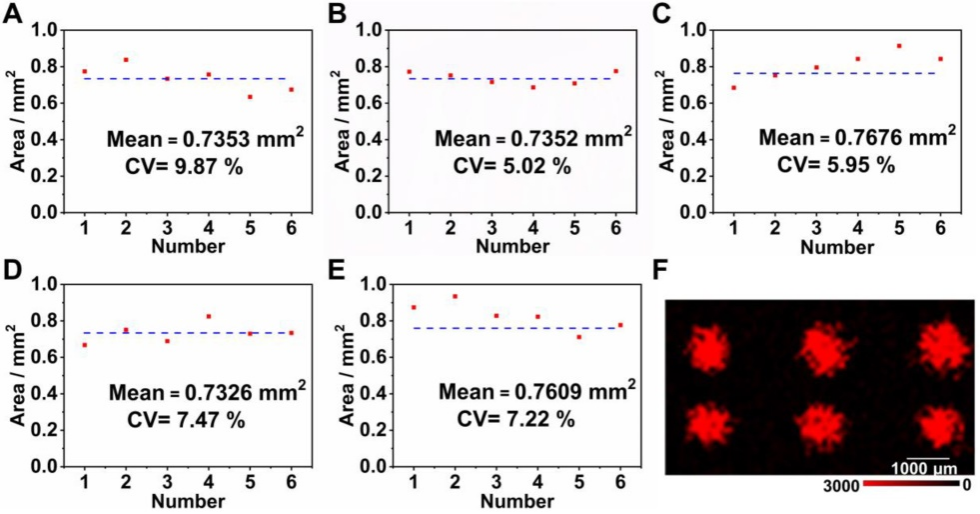
Areas of T dots with the following concentrations of influenza A: (A) 10 pM; (B) 100 pM; (C) 1 nM; (D) 10 nM and (E) 100 nM; (F) Raman mapping image of 10 pM influenza A.

In order to obtain a uniform microarray of suitable dimensions onto the LFM, we firstly used a pipette (0.01-2 μL) to immobilize a 0.01 μL aliquot of capture nucleic acids onto the NC membrane. However, it was practically impossible to precisely align the T dots manually, which was evident in other LFM strips that have been constructed previously 12. The alignment of T dots in a microarray is important in low sensitivity detection in which a color change on the NC membrane cannot be observed by eye and the position for signal detection is dependent on the coordinates of each T dot in the microarray. Furthermore, the original T dot spotted on the NC membrane is approximately 1 mm in diameter and the nucleic acids diffuse during the drying process, enlarging the diameter of the T dots by more than 50%. In this case, adjacent T dots are likely to overlap, leading to cross contamination and signal interference. To solve this problem, we designed a device that combined a micromanipulator system and tapered capillary (with a 100 μm outlet diameter) to ensure precise fabrication of the aligned microarray with each T dot, the diameter of which was approximately 0.5 mm prior to drying. To ensure that the microarray assembled on the NC membranes using a home-made instrument was uniform and sufficiently robust for use in the SERS LFM, a range of concentrations of influenza A was quantified using different arrays and the uniformity of each T dot assessed. Five concentrations of influenza A (10 pM, 100 pM, 1 nM, 10 Nm, and 100 nM) were measured using SERS LFMs. After completion of each assay, a color image of each T dot in the microarray was acquired and analyzed using ImageJ software, taking into account the area of the dots in addition to coefficients of variation (CVs), as shown in Figure 3 (A-E). Mean area was 0.7464 mm^2^ with a CV of 7.1%. These results demonstrate that the microarray fabricated using the home-made instrument was uniform in size. In addition to size uniformity, the uniformity of Raman signal on each T dot is an important consideration because it greatly influences the validity of pathogen quantification, with low target concentrations leading to low Raman signal uniformity 14, 18, 23-25. An image of Raman mapping of influenza A at 10 pM is shown in Figure 3F. The SERS LFM exhibited high Raman signal uniformity 26, indicating that the home-made system was sufficiently robust for microarray fabrication on the NC membrane in a SERS LFM. The results may inspire future potential protein or nucleic acid microarrays for other analytical purposes on chips which are low cost and convenient to use. Furthermore, because sample consumption is low, it is suitable for applications where proteins or nucleic acids are available only in low volumes.

### Optimization of hybridization conditions on SERS LFM

To achieve ultrasensitive detection of nucleic acids from RTI pathogens using the SERS LFM, various experimental parameters which influence the sensitivity, accuracy and reproducibility of the SERS LFM require testing.

**Figure 4:**
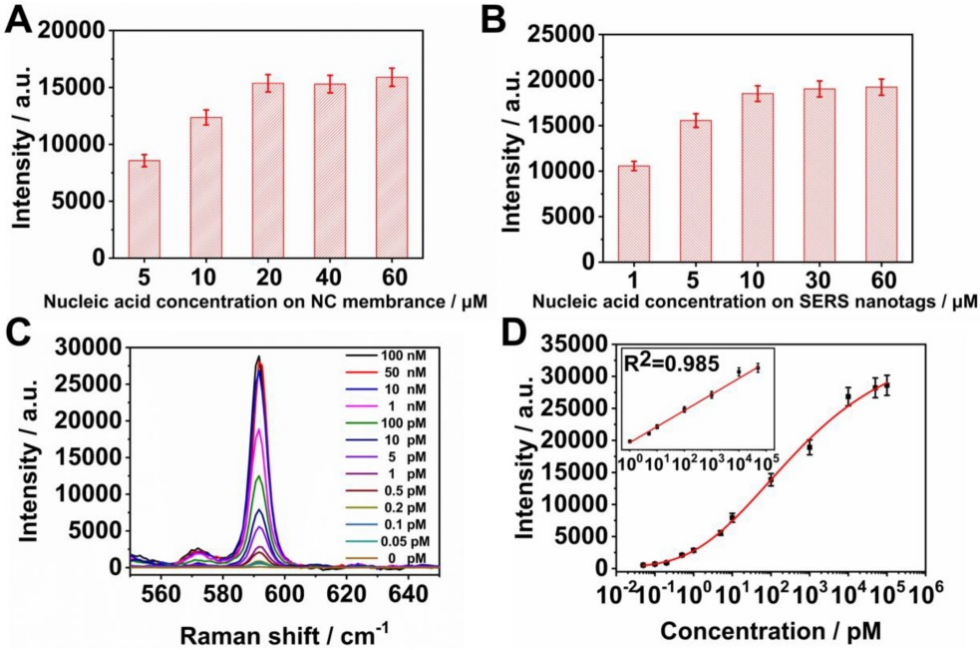
(A) Raman intensity of T dots at various influenza B capture nucleic acid concentrations immobilized on the NC membrane; (B) Raman intensity of T dots with varying concentrations of influenza B detection nucleic acid conjugated to the SERS nanotags; (C) Averaged Raman spectra for different concentrations of influenza A detected by SERS LFM (three repeats); (D) Corresponding calibration lines of influenza A. Error bars represent standard deviations calculated from three measurements.

In the SERS LFM, Raman intensities of the microarrays and C line depended on the quantity of SERS nanotags captured at these positions which in turn correspond to the quantity of detection nucleic acid conjugated to the SERS nanotags and capture nucleic acids immobilized on the NC membrane. Influenza B nucleic acid was chosen as a representative pathogen of the eleven RTI pathogens used in the SERS LFM. Various concentrations of capture nucleic acids were immobilized on the microarrays to ascertain the optimum concentration. A fixed concentration of influenza B of 10 μM was coupled to Ag^MB^@Au NPs and then 100 μL of 100 pM influenza B target nucleic acid placed on the sample pad and the resulting Raman intensity quantified. It can be seen from Figure 4A that Raman intensity increased with increasing nucleic acid concentration from 5 μM to 20 μM. Raman intensity was no higher for immobilized nucleic acid concentrations increased to 40 μM and 60 μM. Taking Raman intensity and cost into consideration, 20 μM immobilized nucleic acid provided the most favorable signal intensity.

To determine the optimum concentration of detection nucleic acids conjugated to Ag@Au NPs, influenza B coupled to Ag^MB^@Au NPs was examined. The concentration of capture nucleic acid of influenza B immobilized on the NC membrane was fixed at 20 μM. As before, 100 μL of 100 pM influenza B target nucleic acid were placed on the sample pad and the resulting Raman intensity quantified. As can be seen from Figure 4B, the Raman intensity increased with increasing nucleic acid concentration from 1 μM to 10 μM. At nucleic acid concentrations of 30 μM and 60 μM, Raman intensity was no higher than it was at 10 μM. Therefore, a detection nucleic acid concentration of 10 μM was chosen for conjugation on the SERS nanotags.

### Detection of influenza A with SERS LFM

To evaluate the performance of the SERS LFM using optimized conditions for ultrasensitive quantification of nucleic acid, strips were first employed to detect various concentrations of influenza A target nucleic acid. Quantitative analysis was conducted by measuring the Raman intensities of the Ag^NBA^@Au SERS nanotags of specific T dots in the microarrays. Mean SERS spectra were calculated to obtain credible results (Figure 4C) and a calibration curve for influenza A obtained (Figure 4D). Raman intensity at 592 cm^-1^ gradually increased as influenza A target nucleic acid concentration was increased. Four parameter logistic fitting models were used to obtain fitting parameters. The inset in the graph demonstrates curve linearity for influenza A (R^2^=0.985). The LOD for influenza A was calculated to be 0.025 pM, which is 5 orders of magnitude smaller than that of gold NP-based LFA 14. The high SVR (surface area to volume ratio) of the NC membrane employed in the SERS LFM assists in expanding the quantitative detection range, the linear dynamic range (LDR) of influenza A spanning 5 orders of magnitude from 1 pM to 50 nM 18, 21. The results demonstrate that the SERS LFM is capable of detecting target pathogen nucleic acids with ultrahigh sensitivity, a wide LDR while requiring less analysis time, and at low-cost.

### Cross reactivity of the SERS LFM

**Figure 5:**
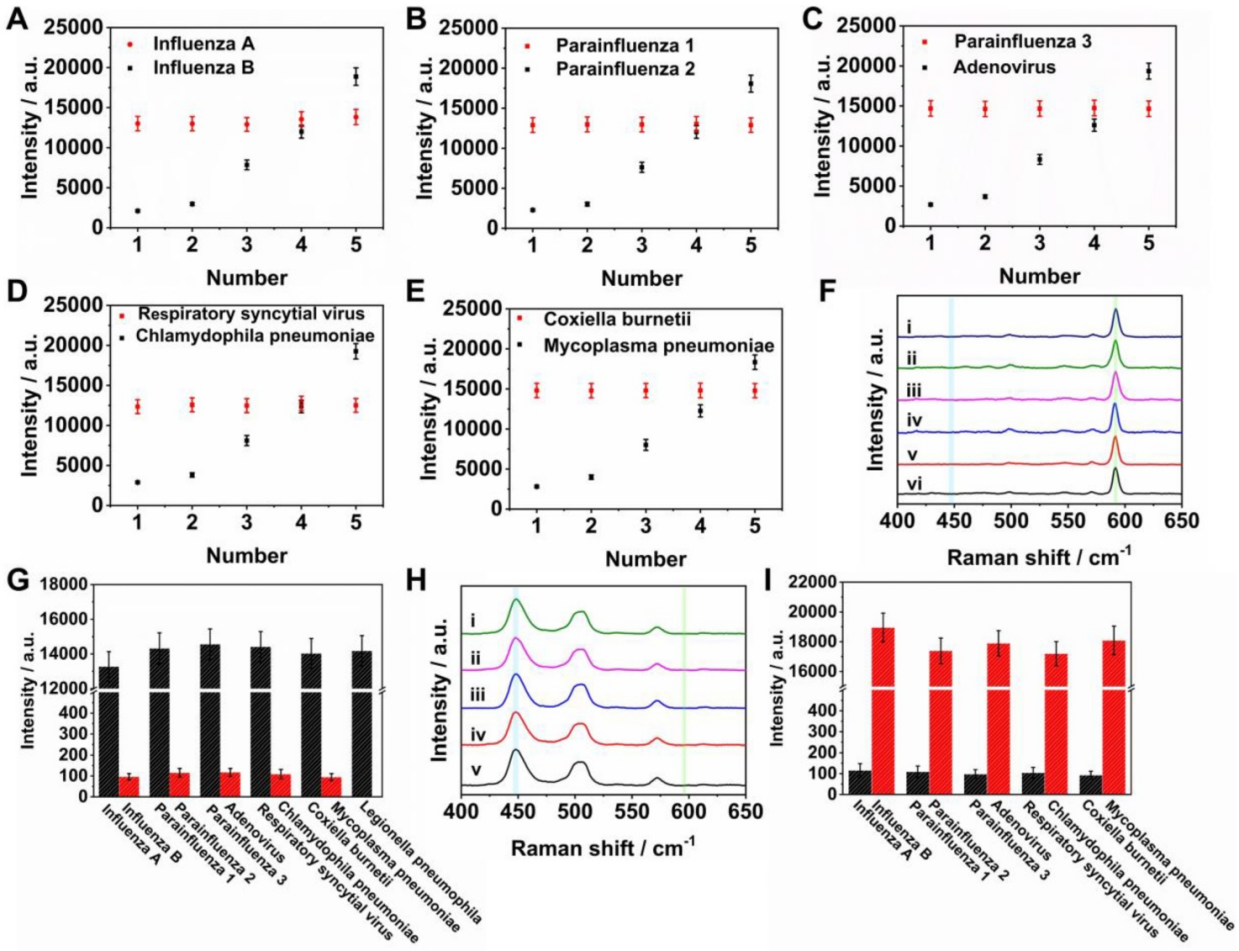
(A-E) Variations in Raman intensity of T dots at 448 and 592 cm^-1^ for different concentrations of influenza A, influenza B, parainfluenza 1, parainfluenza 2, parainfluenza 3, adenovirus, respiratory syncytial virus, chlamydophila pneumoniae, coxiella burnetii and mycoplasma pneumoniae; (F) Raman spectra: (ⅰ) corresponds to T dot detection of influenza A and influenza B; (ⅱ) corresponds to T dot detection of parainfluenza 1 and parainfluenza 2; (ⅲ) corresponds to T dot detection of parainfluenza 3 and adenovirus; (ⅳ) corresponds to T dot detection of respiratory syncytial virus and chlamydophila pneumoniae; (ⅴ) corresponds to T dot detection of coxiella burnetii and mycoplasma pneumoniae; (ⅵ) corresponds to T dot detection of legionella pneumophila; (H) Raman spectra: (ⅰ) corresponds to T dot detection of influenza A and influenza B; (ⅱ) corresponds to T dot detection of parainfluenza 1 and parainfluenza 2; (ⅲ) corresponds to T dot detection of parainfluenza 3 and adenovirus; (ⅳ) corresponds to T dot detection of respiratory syncytial virus and chlamydophila pneumoniae; (ⅴ) corresponds to T dot detection of coxiella burnetii, and mycoplasma pneumoniae; (G, I) Variations in Raman intensity of T dots at 448 and 592 cm^-1^ for different concentrations of influenza A, influenza B, parainfluenza 1, parainfluenza 2, parainfluenza 3, adenovirus, respiratory syncytial virus, chlamydophila pneumoniae, coxiella burnetii, mycoplasma pneumoniae, and legionella pneumophila (three repeats).

To assess the cross reactivity of two nucleic acids markers on the same T dot, 100 pM target nucleic acids of influenza A, parainfluenza 1, parainfluenza 3, respiratory syncytial virus and coxiella burnetii were mixed at different concentrations of target nucleic acids of influenza B, parainfluenza 2, adenovirus, chlamydophila pneumoniae and mycoplasma pneumoniae, over the range 0.5 to 500 pM (0.5, 1, 10, 50, and 500 pM). For example, a mixture of influenza A and influenza B (Figures 5A and S2A) resulted in a Raman intensity of Ag^NBA^@Au at 592 cm^-1^ that was practically unchanged for 100 pM influenza A in all samples. The Raman intensity of Ag^MB^@Au at 448 cm^-1^ became stronger as the concentration of influenza B increased from 0.5 to 500 pM, demonstrating that high concentrations of influenza A did not affect the sensitivity of the assay for influenza B. Likewise, tests of cross reactivity between parainfluenza 1 and parainfluenza 2, parainfluenza 3 and adenovirus, respiratory syncytial virus and chlamydophila pneumoniae, and coxiella burnetii with mycoplasma pneumoniae demonstrated the same response, which are shown in Figures 5B-5E and S2B-S2E. The results demonstrate that the cross interference between influenza A and influenza B, parainfluenza 1 and parainfluenza 2, parainfluenza 3 and adenovirus, respiratory syncytial virus and chlamydophila pneumoniae, and coxiella burnetii and mycoplasma pneumoniae was negligible in the SERS LFM. This is due to the specific base-pairing among the capture nucleic acids, detection nucleic acids and target nucleic acids. In addition, the detectable concentration difference between each nucleic acid mixture is as large as 10^4^-fold, a requirement for simultaneous quantification of two nucleic acids on one T dot as concentrations of several orders of magnitude may be simultaneously experienced when an individual is infected by several RTI pathogens.

To estimate the selectivity for multiplex quantification of the nucleic acids of eleven RTI pathogens using the SERS LFM, 0 pM target nucleic acids of influenza A, parainfluenza 1, parainfluenza 3, respiratory syncytial virus, coxiella burnetii and legionella pneumophila mixed with 100 pM target nucleic acid from influenza B, parainfluenza 2, adenovirus, chlamydophila pneumoniae and mycoplasma pneumoniae were measured (Figure 5F). The corresponding Raman intensities are shown in Figure 5G. For the Raman spectra in Figure 5F (ⅰ), corresponding to the T dot for detecting influenza A and influenza B, a SERS signal corresponding to Ag^NBA^@Au-labeled influenza B nucleic acid was observed, indicating successful identification of the specific influenza B target nucleic acid. Similar results were observed in other Raman spectra, Figure 5F (ⅱ, ⅲ, ⅳ, ⅴ, ⅵ). Similarly, 100 pM target nucleic acids of influenza A, parainfluenza 1, parainfluenza 3, respiratory syncytial virus, coxiella burnetii and legionella pneumophila mixed with 0 pM target nucleic acids of influenza B, parainfluenza 2, adenovirus, chlamydophila pneumoniae, and mycoplasma pneumoniae were measured (Figure 5H). The corresponding Raman intensities are shown in Figure 5I. For Raman spectra in Figure 5H (ⅰ), corresponding to the T dot for detection of influenza A and influenza B, the SERS signal corresponding to Ag^MB^@Au labeled influenza A nucleic acid was measured, indicating successful identification of the specific influenza A target nucleic acid. Similar results were also observed in the other Raman spectra in Figure 5H (ⅱ, ⅲ, ⅳ, ⅴ). These results are suggestive of a highly functional and specific sandwich assay providing recognition of SERS signals attributable to the correct nucleic acid.

### Analytical performance of SERS LFM

**Figure 6:**
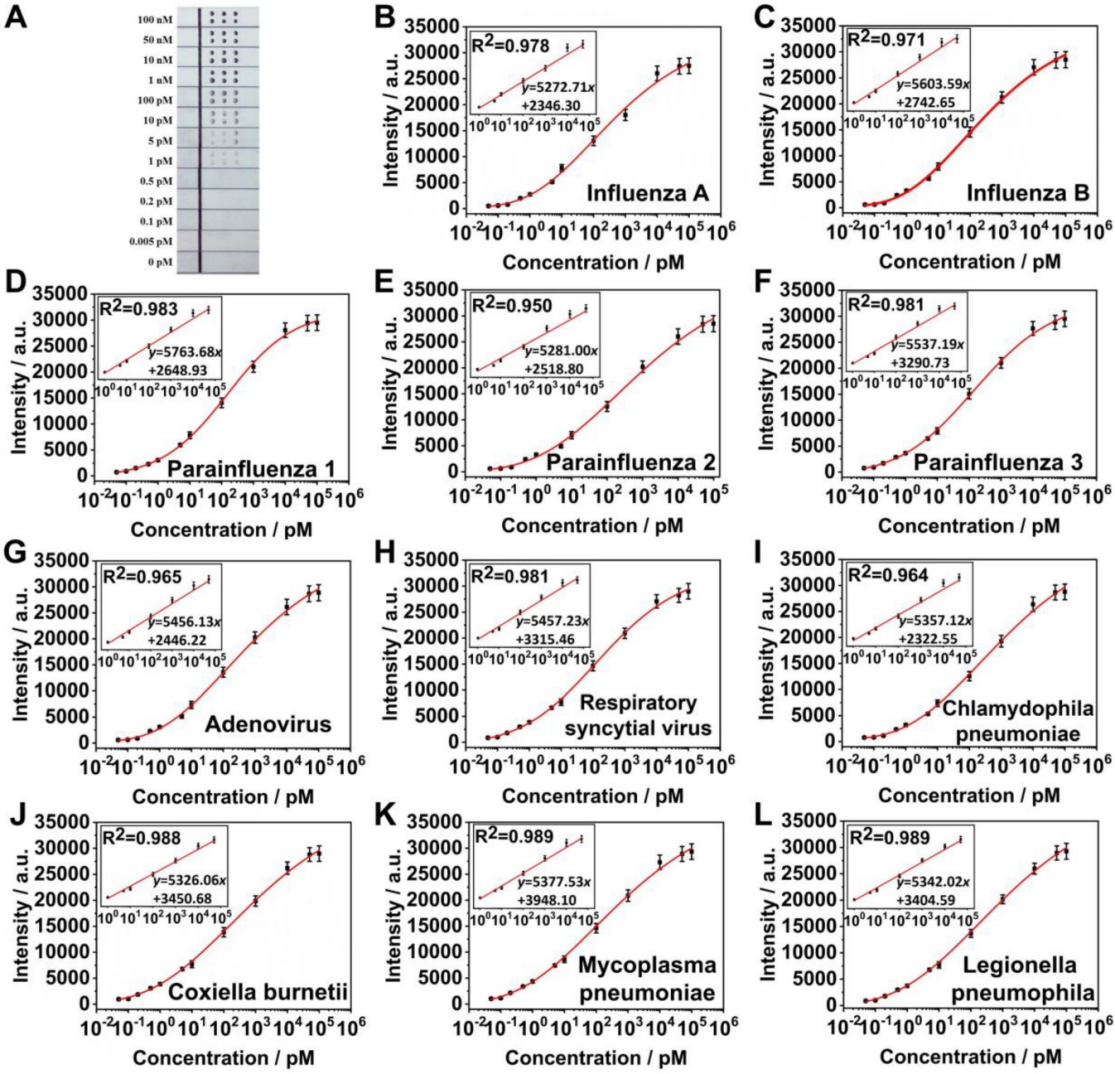
(A) Image of SERS LFM with different concentrations of influenza A, influenza B, parainfluenza 1, parainfluenza 2, parainfluenza 3, adenovirus, respiratory syncytial virus, chlamydophila pneumoniae, coxiella burnetii, mycoplasma pneumoniae and legionella pneumophila (from 0-100 nM). Calibration curves for (B) influenza A, (C) influenza B, (D) parainfluenza 1, (E) parainfluenza 2, (F) parainfluenza 3, (G) adenovirus, (H) respiratory syncytial virus, (I) chlamydophila pneumoniae, (J) coxiella burnetii, (K) mycoplasma pneumoniae, and (L) legionella pneumophila. Graphs represent three repeats of measurements of each pathogen. Insets display the linear part of the calibration curve.

To evaluate the capability of the SERS LFM to perform ultrasensitive and multiplex quantification of nucleic acids, the system was tested by measuring the target nucleic acids of eleven RTI pathogens at varying concentrations. Figure 6A displays an image of SERS LFMs testing target nucleic acids of various concentrations of eleven RTI pathogens. The visual LOD of the target nucleic acids of all eleven RTI pathogens were 1 pM. Quantitative detection was conducted through inspection of the Raman intensities of the Ag^NBA^@Au and Ag^MB^@Au SERS nanotags of every T dot. Mean SERS spectra were calculated to obtain accurate results (Figure S3), with Raman intensities increasing progressively as RTI pathogen target nucleic acid concentration increased, allowing plotting of calibration curves (Figures 6B-6L). Fitting parameters were calculated through applying a four-parameter logistic fitting model. Insets display the formulae of calibration curves and their linear section for influenza A, parainfluenza 1, parainfluenza 3, respiratory syncytial virus, coxiella burnetii, and legionella pneumophila, influenza B, parainfluenza 2, adenovirus, chlamydophila pneumoniae and mycoplasma pneumoniae. The LOD for influenza A, parainfluenza 1, parainfluenza 3, respiratory syncytial virus, coxiella burnetii, legionella pneumophila, influenza B, parainfluenza 2, adenovirus, chlamydophila pneumoniae and mycoplasma pneumoniae were calculated to be 0.031 pM, 0.030 pM, 0.038 pM, 0.038 pM, 0.040 pM, 0.039 pM, 0.035 pM, 0.032 pM, 0.040 pM, 0.039 pM, and 0.041 pM, respectively. It can be seen that the LOD for influenza A increased slightly when influenza A and influenza B were detected on one T dot (0.031 pM) compared to when influenza A was on the T dot alone (0.025 pM). The reason relates to the surface area of the NC membrane being occupied by a greater number of SERS nanotags with the SVR being divided by the number of different SERS probe categories 18, 21. The limit of quantification (LOQ) for influenza A, parainfluenza 1, parainfluenza 3, respiratory syncytial virus, coxiella burnetii, legionella pneumophila, influenza B, parainfluenza 2, adenovirus, chlamydophila pneumoniae, and mycoplasma pneumoniae were calculated to be 0.157 pM, 0.149 pM, 0.261 pM, 0.219 pM, 0.326 pM, 0.256 pM, 0.195 pM, 0.167 pM, 0.272 pM, 0.353 pM, and 0.351 pM, respectively. The LDR of measurement of the target nucleic acids of the eleven RTI pathogens were 1 pM-50 nM, which span 5 orders of magnitude. A comparison of LDRs, detection time, LODs and the number of targets among the LFAs/LFM with various labels are shown in Table S1. The results demonstrate that the LOD of the SERS LFM is significantly lower than other LFM/LFAs, while the respective LDR is wider. Additionally, the time required for detection is relatively short for quantification of the target nucleic acids of the eleven RTI pathogens. It requires approximately 20 min (7 min for the reaction, 13 min for measuring the Raman signal) to obtain quantitative results for eleven nucleic acids, considerably accelerating the screening of RTI pathogens compared with other technologies. The SERS LFM achieves a CV (coefficient of variation) as low as 9.5% across different batches. Furthermore, the capacity of the SERS LFM to provide biological results was greatly enhanced by adoption of both the encoded SERS nanotags 27-30 and microarray. In addition, miniaturization of the T lines to the T dots in the microarrays for LFA greatly decreased sample volumes, reagent consumption, requirement for manual operation and the time required for detection. All these demonstrate that the SERS LFM can be used for ultrasensitive and rapid quantification of the target nucleic acids of eleven RTI pathogens with ultrahigh sensitivity, high detection throughput, wide LDR, low cost, low detection time that is easy to use in addition to providing excellent reproducibility. The device will provide considerable capability for clinical on-site investigation and personalized healthcare. It also provides a method which can greatly enhance the application of LFM in many other locations, including consulting rooms, clinics and even in space.

High accuracy requires not only high precision, but also high trueness. For the sake of evaluating the reliability, accuracy and the feasibility of using the SERS LFM in a clinical investigation, three different concentrations of target nucleic acids of RTI pathogens were added to a blank human throat swab sample and then analyzed using the SERS LFM (Figure S4). The results demonstrate that the percentage recovery of influenza A, parainfluenza 1, parainfluenza 3, respiratory syncytial virus, coxiella burnetii, legionella pneumophila, influenza B, parainfluenza 2, adenovirus, chlamydophila pneumoniae, and mycoplasma pneumoniae ranged from 98.6% to 103.7%, 93.5% to 106.1%, 96.6% to 107.9%, 93.9% to 104.3%, 95.8% to 107.3%, 96.9% to 103.6%, 95.6% to 106.7%, 97.1% to 104.7%, 91.4% to 95.8%, 97.6% to 105.9%, and 92.7% to 108.7% (Table S2), respectively, which is within the range required for correct investigation, demonstrating the trueness of the SERS LFM. Mean CV was calculated to be 8.2%, demonstrating that the SERS LFM exhibited excellent precision. Thus, the high trueness and precision of the SERS LFM ensures highly accurate output imperative for correct diagnosis of the target nucleic acids of RTI pathogens. The original SERS LFA that was fabricated 14, 18 is characterized by being low cost, sufficiently small to store and transport, user-friendly, not requiring any expertise and delivering results quickly during clinical usage. The current SERS LFM has added additional versatility by increasing simultaneous detection of eleven RTI pathogens per strip, which is more than three times greater than the original SERS LFA. In addition, the cost of materials, reagent consumption, preparation and detection time, in addition to the time required for experimental procedures and operation are greatly reduced compared with the SERS LFA, using a compact and integrative design. The SERS LFM provides an accurate and rapid diagnosis of RTI pathogens at the point of care, which will greatly assist in the timely and accurate diagnosis of RTI and for selecting an individual treatment plan. It will also standardize the use of antibiotics. A confocal Raman microscope system for research was employed to discover the association between Raman signal intensity, sample concentration and signal homogeneity across the T dots in the SERS LFM. This system offers a powerful reference for portable Raman devices, which will become increasingly available over the coming years 31 and indicates the future potential of diagnostic applications at community health centers and hospitals in addition to within poor areas with restricted resources.

## Conclusions

In this study, a SERS LFM that achieves ultrasensitive and high throughput diagnosis of the nucleic acids of RTI pathogens was described. Core shell SERS nanotags encoded with two RDs were chosen as signal labels and combined on a microarray immobilized on a NC membrane for rapid quantification of the nucleic acids from eleven RTIs on a single strip. The volume of sample required, reagent consumption, material cost, duration of assay preparation and detection, in addition to operation of the test have been reduced because the multiplex assays have been integrated onto one SERS LFM. The LOD for influenza A, parainfluenza 1, parainfluenza 3, respiratory syncytial virus, coxiella burnetii, legionella pneumophila, influenza B, parainfluenza 2, adenovirus, chlamydophila pneumoniae, and mycoplasma pneumoniae were 0.031 pM, 0.030 pM, 0.038 pM, 0.038 pM, 0.040 pM, 0.039 pM, 0.035 pM, 0.032 pM, 0.040 pM, 0.039 pM, and 0.041 pM, respectively. The LOQ for influenza A, parainfluenza 1, parainfluenza 3, respiratory syncytial virus, coxiella burnetii, legionella pneumophila, influenza B, parainfluenza 2, adenovirus, chlamydophila pneumoniae, and mycoplasma pneumoniae were 0.157 pM, 0.149 pM, 0.261 pM, 0.219 pM, 0.326 pM, 0.256 pM, 0.195 pM, 0.167 pM, 0.272 pM, 0.353 pM, and 0.351 pM, respectively. The LDR of these eleven RTI pathogen nucleic acids were 1 pM-50 nM, which cover 5 orders of magnitude. The test benefitted from signal amplification of the encoded SERS nanotags and the 2×3 microarray embedded on the NC membrane, and the high SVR of the NC membrane. It is foreseeable that SERS LFM technology could in future be the platform for high throughput RTI nucleic acid detection with ultra-high sensitivity, broad LDR at low-cost with a short detection time, combined with simple manual operation, providing potential applications in personalized healthcare.

## Figures and Tables

**Table 1:** Nucleic acids sequences used.

Name	Sequences (5' to 3')	Length	Label
Influenza A-target:	CATTTTGGACAAAGCGTCTACGCTGCAGTCCTCGCTCACTGG	42	
Influenza A-detection:	TTTTTTTTTTGCTCACCGTGCCCAGTGAGCGAGGAC	36	5'Amino Modifier C12
Influenza A-capture:	TAGACGCTTTGTCCAAAATGCCCTATTTTTTTTTT	35	3'Biotin
Influenza B-target:	TAGTAAGGCTTGCTTTTATTTAATCCACCGTATTTTTCAT	40	
Influenza B-detection:	TTTTTTTTTTATTGCCTCCATGAAAAATACGGTGG	35	5'Amino Modifier C12
Influenza B-capture:	AAAAGCAAGCCTTACTACACAGGGGTTTTTTTTTT	35	3'Biotin
Parainfluenza 1-target:	GTCATTGATGTCATAGGTATGAGAAATTACCGGGTTTAAATC	42	
Parainfluenza 1-detection:	TTTTTTTTTTATGTATCCTGATTTAAACCCGGTAA	35	5'Amino Modifier C12
Parainfluenza 1-capture:	ATACCTATGACATCAATGACAACAGTTTTTTTTTT	35	3'Biotin
Parainfluenza 2-target:	CCTGAAGATTGGAAATGCTGCAGCAGATTGTTGTATTATCC	41	
Parainfluenza 2-detection:	TTTTTTTTTTTAGCAATGGGGATAATACAACAATC	35	5'Amino Modifier C12
Parainfluenza 2-capture:	AGCATTTCCAATCTTCAGGACTATGATTTTTTTTTT	36	3'Biotin
Parainfluenza 3-target:	CAGTCGTTGGCATAGCTAATAATCCTGGTCCCGGCATTAAT	41	
Parainfluenza 3-detection:	TTTTTTTTTTAAAAATAAGATTAATGCCGGGACCAG	36	5'Amino Modifier C12
Parainfluenza 3-capture:	TAGCTATGCCAACGACTGTTGATGGCTTTTTTTTTT	36	3'Biotin
Adenovirus-target:	CTCCGAGGCGTCCTGCCCGGAGATGTGCATGTAAGACCACT	41	
Adenovirus-detection:	TTTTTTTTTTGCCCCAGTGGTCTTACATGCACA	33	5'Amino Modifier C12
Adenovirus-capture:	GGCAGGACGCCTCGGAGTACCTGAGCTTTTTTTTTT	36	3'Biotin
Respiratory syncytial virus-target:	TTGTTGAGTGTATCATTCAACTTGACTTTGCTAAGAGCCAT	41	
Respiratory syncytial virus-detection:	TTTTTTTTTTGCAAATACAAAAATGGCTCTTAGCAA	36	5'Amino Modifier C12
Respiratory syncytial virus-capture:	GTTGAATGATACACTCAACAAAGATTTTTTTTTTT	35	3'Biotin
Chlamydophila pneumoniae-target:	TTGTGGTTGTGTAGTCTGAGGTGTTTGTGCTACTGTAGCCAT	42	
Chlamydophila pneumoniae-detection:	TTTTTTTTTTAGAACAACATGGCTACAGTAGCACAA	36	5'Amino Modifier C12
Chlamydophila pneumoniae-capture:	GACTACACAACCACAACCATCAGTATCTTTTTTTTTT	37	3'Biotin
Coxiella burnetii-target:	GTTATTGCAACTTTAGAAGCTGCTGTTTTAAAGGCAGTAGT	41	
Coxiella burnetii-detection:	TTTTTTTTTTACAGACGGTACTACTGCCTTTAAAAC	36	5'Amino Modifier C12
Coxiella burnetii-capture:	CTTCTAAAGTTGCAATAACCCAAAACTTTTTTTTTT	36	3'Biotin
Mycoplasma pneumoniae-target:	TTTCATTCTTGCGAATGTACTACCCAGGCGAGATACTTAA	40	
Mycoplasma pneumoniae-detection:	TTTTTTTTTTTAACACATTAAGTATCTCGCCTGGG	35	5'Amino Modifier C12
Mycoplasma pneumoniae-capture:	CATTCGCAAGAATGAAACTCAAACGTTTTTTTTTT	35	3'Biotin
Legionella pneumophila-target:	GTTAAAAAGGCTTCCCCTTTTACTTTATTTTCATCCGCTT	40	
Legionella pneumophila-detection:	TTTTTTTTTTGAATTCAATAAGAAAGCGGATGAAAATA	38	5'Amino Modifier C12
Legionella pneumophila-capture:	AAGGGGAAGCCTTTTTAACTGAAAACATTTTTTTTTT	37	3'Biotin
Control nucleic acid	TTTTTTTTTTTGCATCCACCAGCAGTAACTCCCCACAA	38	5'Amino Modifier C12
Complementary chain of control nucleic acid	TTTTTTTTTTAAAGAGGTTGTGGGGAGTTACTGCTGG	37	3'Biotin

## Abbreviations

SERS: surface enhanced Raman scattering
RTI: respiratory tract infection
LOD: limit of detection
LOQ: limit of quantification
LDR: linear dynamic range
NC: nitrocellulose
SVR: surface area to volume ratio
LFM: lateral flow microarray
LFA: lateral flow assay
C: control
T: test
RD: Raman dye
POCT: point of care testing
LRTI: lower respiratory tract infection
URTI: upper respiratory tract infection
IFA: indirect immunofluorescence assay
IVD: *in vitro* diagnostic
NBA: Nile blue A
MB: methylene blue
NP: nanoparticle
CV: coefficient of variation
