# Supplementary material for: Rapid and Ultrasensitive Quantification of Multiplex Respiratory Tract Infection Pathogen via Lateral Flow Microarray based on SERS Nanotags
Source: Theranostics. 2019 Jul 9;9(17):4849–59. doi: 10.7150/thno.35824 (PMC6691375; doi:10.7150/thno.35824)
Supplement: Supplementary file 1 — Supplementary figures and tables. [file thnov09p4849s1.pdf]

Supplementary Material

Rapid and ultrasensitive quantification of multiplex respiratory tract infection pathogen via lateral flow microarray based on SERS nanotags

Di Zhang[1,2,3], Li Huang[1,2,4], Bing Liu[1,2], Qinyu Ge[1,2], Jian Dong[1,2] and Xiangwei Zhao[*,1,2]

1. State Key Laboratory of Bioelectronics, School of Biological Science and Medical Engineering, Southeast University, Nanjing 210096, China
2. National Demonstration Center for Experimental Biomedical Engineering Education, Southeast University, Nanjing 210096, China
3. Department of Biomedical Engineering, Yale University, New Haven, CT 06520, USA
4. Getein Biotechnology Co., Ltd., Nanjing 210000, China

[*] Corresponding to: State Key Laboratory of Bioelectronics, School of Biological Science and Medical Engineering, Southeast University, Nanjing 210096, China E-mail: xwzhao@seu.edu.cn (X Zhao)

Table of contents

SERS measurement........S2
Figure S1........S3
Figure S2........S4
Figure S3........S5
Figure S4........S6
Table S1........S7
Table S2........S8
References........S9

Experimental

SERS measurement

Raman spectra of SERS nanotag and T dots of the SERS LFM strip as well as Raman mapping images were acquired using an In Via Renishaw Raman microscope system (Renishaw, New Mills, UK). The laser used was a 785 nm line source. Baseline correction of each Raman spectrum was performed using Renishaw Wire 4.2 software, and the baseline was corrected as zero. In this work, a 20× objective lens with the numerical aperture of 0.4 was used. For Ag^{MB}@Au NPs and Ag^{NBA}@Au, 10 μL of NPs were transferred to a capillary tube, the Raman spectra were measured by focusing a laser dot on the tube. The acquisition time was 10 s. The corresponding characteristic Raman shifts of MB and NBA are at 448, and 592 cm^{-1}, respectively. Raman spectra and Raman mapping images for the T dots of SERS LFM strips were measured using 785 nm laser and integration time was 1 s. A holographic notch filter was placed in the collection path to remove the Rayleigh line from the collected Raman data. The spectral resolution can reach 1 cm^{-1}.

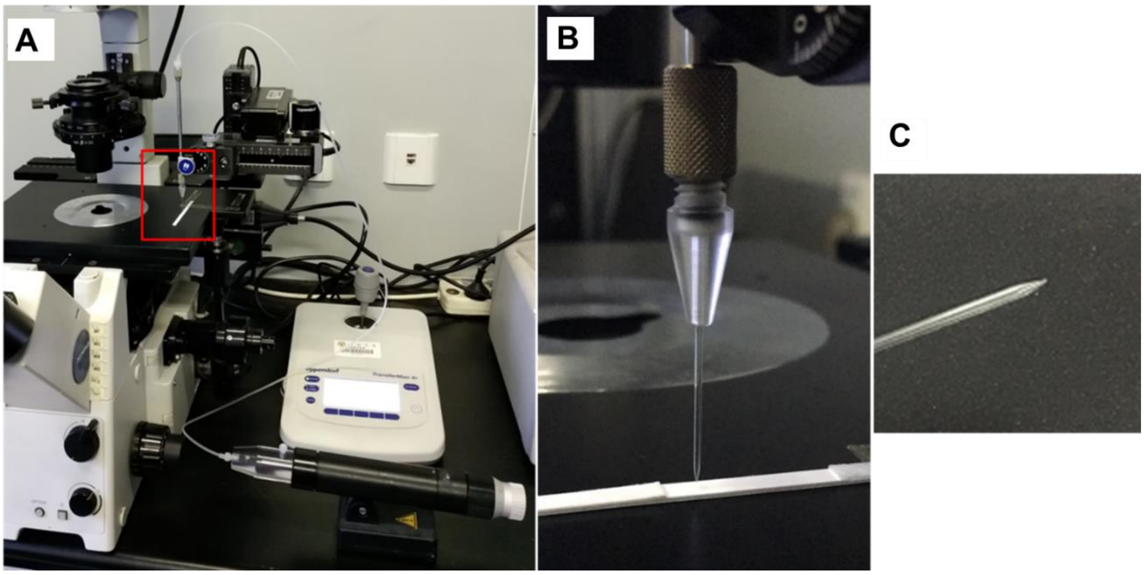

Figure S1. (A) The micromanipulator system for microarray fabrication; (B) Enlarged view of the red box in (A); (C) The capillary with tapered head.

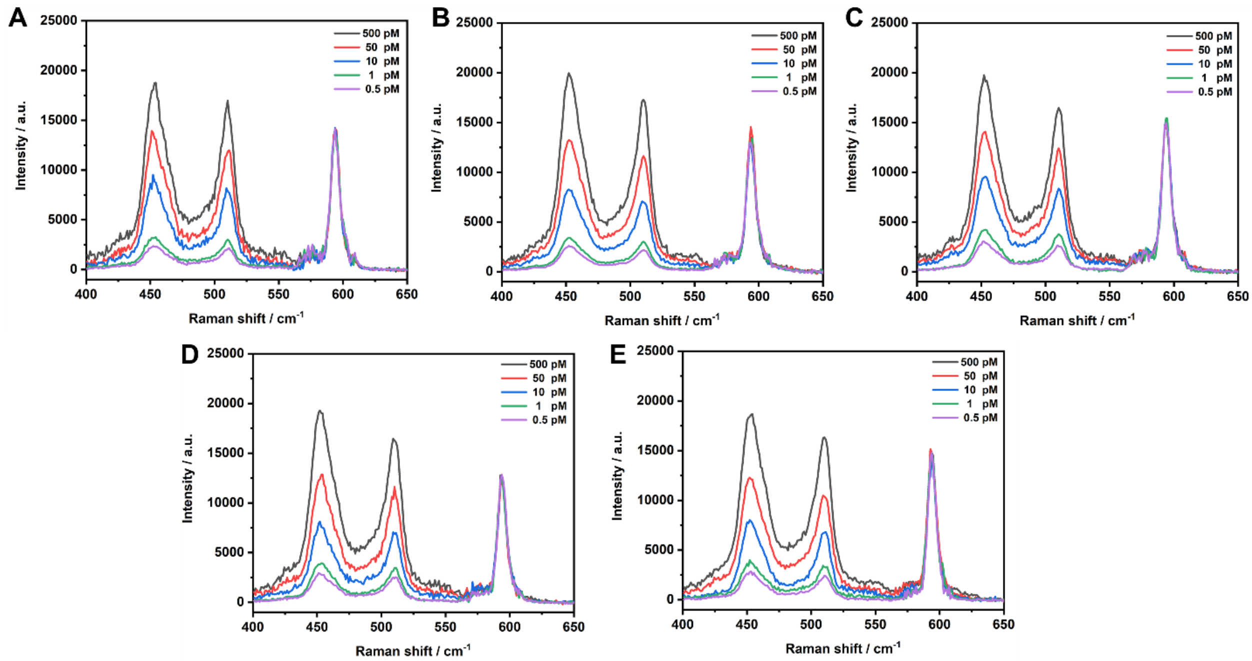

Figure S2. Averaged Raman spectra of T dots with different concentrations of (A) influenza A (100 pM) and influenza B (0.5, 1, 10, 50, 500 pM); (B) parainfluenza 1 (100 pM) and parainfluenza 2 (0.5, 1, 10, 50, 500 pM); (C) parainfluenza 3 (100 pM) and adenovirus (0.5, 1, 10, 50, 500 pM); (D) respiratory syncytial virus (100 pM) and chlamydophila pneumoniae (0.5, 1, 10, 50, 500 pM); (E) coxiella burnetii (100 pM) and mycoplasma pneumoniae (0.5, 1, 10, 50, 500 pM).

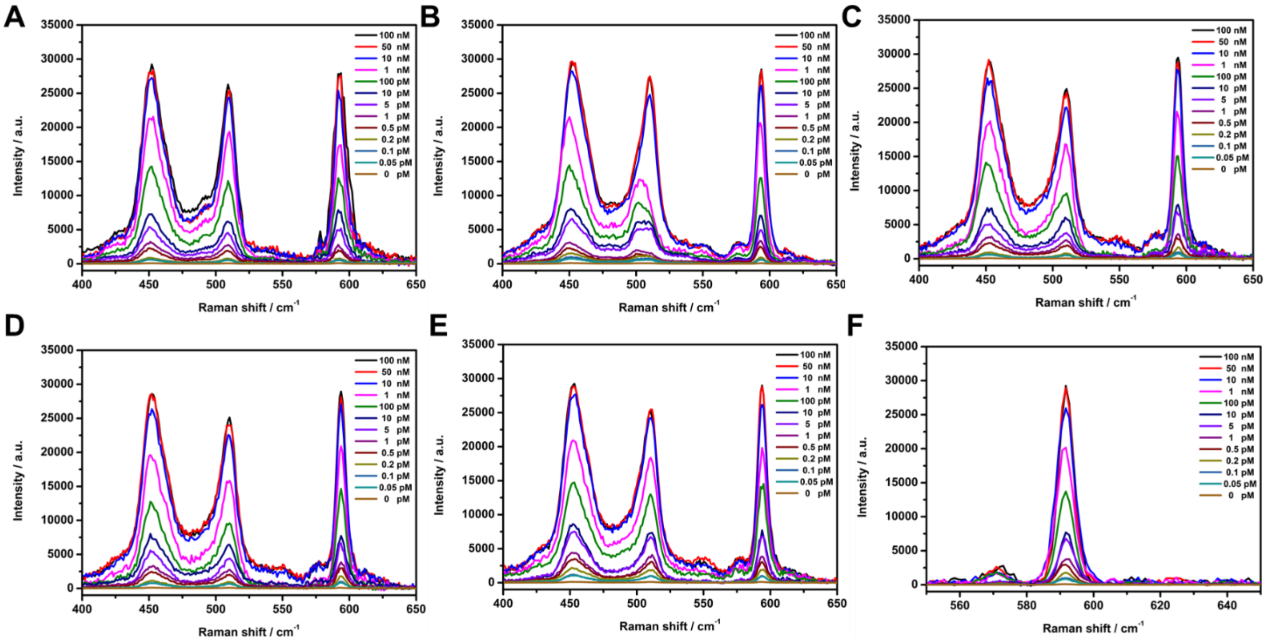

Figure S3. Averaged Raman spectra of the T dots under different concentrations of influenza A and influenza B (A), parainfluenza 1 and parainfluenza 2 (B), parainfluenza 3 and adenovirus (C), respiratory syncytial virus and chlamydophila pneumoniae (D), coxiella burnetiid and mycoplasma pneumoniae (E), and legionella pneumophila (F).

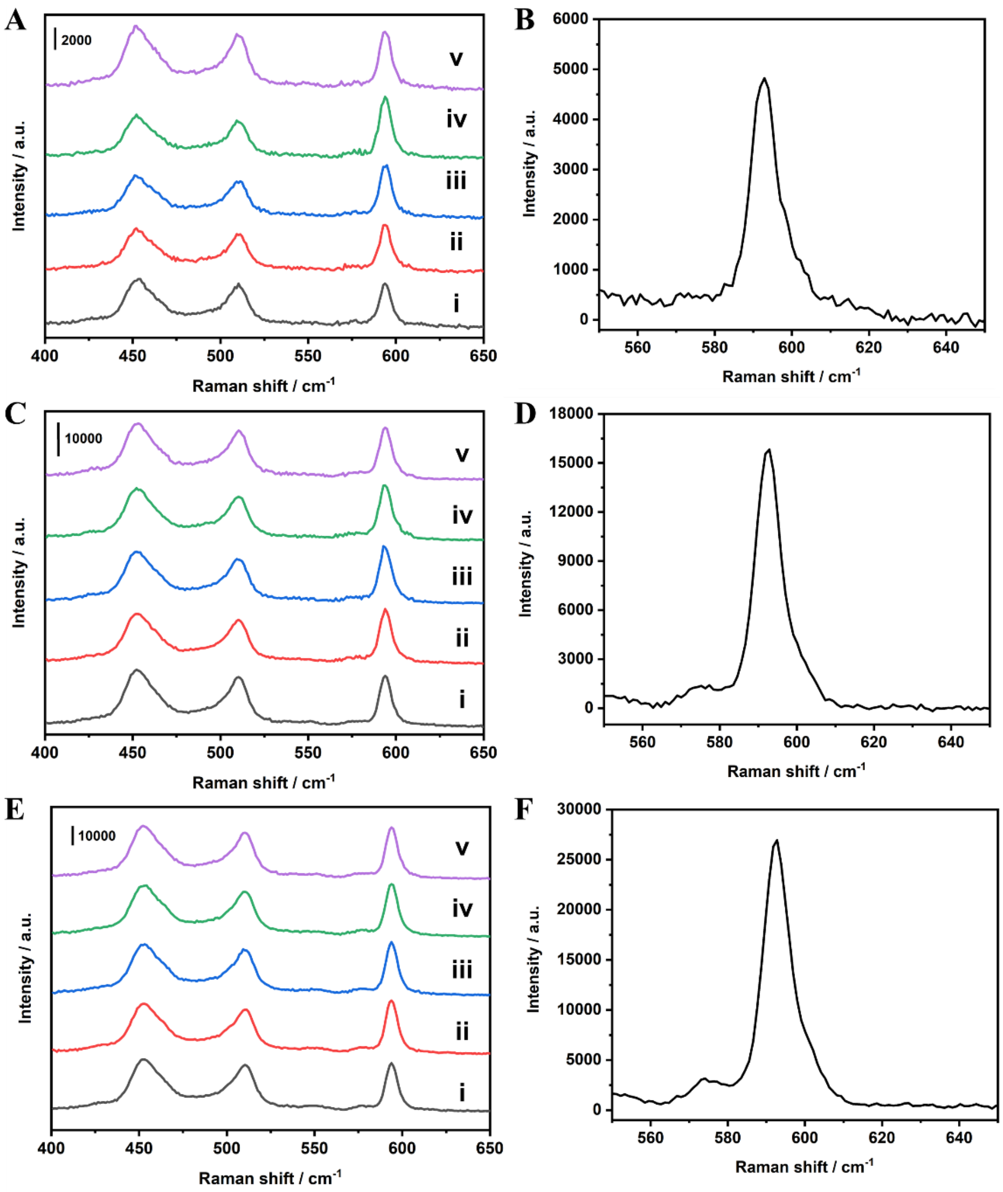

Figure S4. Averaged Raman spectra with various concentrations of RTI pathogen target nucleic acids added in blank human throat swab sample. (A) 2 pM influenza A and influenza B (i), parainfluenza 1 and parainfluenza 2 (ii), parainfluenza 3 and adenovirus (iii), respiratory syncytial virus and chlamydophila pneumoniae (iv), coxiella burnetiid and mycoplasma pneumoniae (v), and (B) legionella pneumophila. (C) 200 pM influenza A and influenza B(i), parainfluenza 1 and parainfluenza 2 (ii), parainfluenza 3 and adenovirus (iii), respiratory syncytial virus and chlamydophila pneumoniae (iv), coxiella burnetiid and mycoplasma pneumoniae (v), and (D) legionella pneumophila. (E) 20000 pM influenza A and influenza B (i), parainfluenza 1 and parainfluenza 2 (ii), parainfluenza 3 and adenovirus (iii), respiratory syncytial virus and chlamydophila pneumoniae (iv), coxiella burnetiid and mycoplasma pneumoniae (v), and (F) legionella pneumophila.

Table S1. Comparison of limit of detections, linear dynamic ranges, number of targets, and detection time among LFAs with different labels for nucleic acids detection.

No.	Analysts	labels	Limit of detection (LOD)	Amplification method	Linear dynamic range (LDR)	Number of targets	Time	Reference
1	HIV-1	Au NPs	0.1 nM	No		1	20 min	S1
2	Multiplex blood group genotyping	Au NPs	Qualitative detection	LATE-PCR		8	60 min	S2
3	DNA	Carbon nanotube	40 pM	No	0.1-20 nM	1	20 min	S3
4	Kaposi's sarcoma-associated herpesvirus (KSHV) DNA and bacillary angiomatosis (BA) DNA	SERS nanotags	KSHV: 0.043 pM, BA: 0.074 pM	No		2	20 min	S4
5	Genotyping of seven pathogenic single nucleotide polymorphisms in phenylalanine hydroxylase gene	Gold magnetic nanoparticles (GMNPs)	0.04 pg/μL with plasmid	Amplification refractory mutation system (ARMS) polymerase chain reaction	0.02 to 2 pg/μL		22 min	S5
6	Multiplex respiratory tract infection virus nucleic acids	Encoded SERS nanotags	Influenza A: 0.031 pM, Parainfluenza 1: 0.030 pM, parainfluenza 3: 0.038 pM, respiratory syncytial virus: 0.038 pM, coxiella burnetii: 0.040 pM, legionella pneumophila: 0.039 pM, influenza B: 0.035 pM, parainfluenza 2: 0.032 pM, adenovirus: 0.040 pM, chlamydophila pneumoniae: 0.039 pM, mycoplasma pneumoniae: 0.041 pM	No	1 pM-50 nM	11	~20 min	This work

Table S2. Detection of spiked throat swab samples.

Nucleic acids	Concentration / pM		R^c / %
	Spiked[a]	SERS LFA[b]	
Influenza A	2	1.971±0.117	98.6
	200	203.62 ± 8.474	101.8
	20000	20740±1328	103.7
Influenza B	2	1.912±0.119	95.6
	200	199.21±9.763	99.6
	20000	21340±1198	106.7
Parainfluenza 1	2	2.044±0.121	102.2
	200	212.11±11.982	106.1
	20000	18702±1034	93.5
Parainfluenza 2	2	1.964±0.115	98.2
	200	194.2±8.839	97.1
	20000	20940±1095	104.7
Parainfluenza 3	2	2.158±0.123	107.9
	200	205.47±9.376	102.7
	20000	19320±927	96.6
Adenovirus	2	1.916±0.119	95.8
	200	182.8±10.819	91.4
	20000	18860±1083	94.3
Respiratory syncytial virus	2	2.086±0.112	104.3
	200	187.8±12.253	93.9
	20000	21840±1193	109.2
Chlamydophila pneumoniae	2	1.952±0.117	97.6
	200	211.8±10.829	105.9
	20000	21160±1278	105.8
Coxiella burnetii	2	1.970±0.120	98.5
	200	191.6±9.928	95.8
	20000	21460±975	107.3
Mycoplasma pneumoniae	2	2.174±0.123	108.7
	200	185.4±11.342	92.7
	20000	20480±1036	102.4
Legionella pneumophila	2	1.974±0.115	98.7
	200	193.8±8.626	96.9
	20000	20720±1126	103.6

[a]The RTI pathogen target nucleic acids spiked in real sample.

[b]The average value was calculated based on three repeats for each sample.

[c]R stands for recovery.

References

[S1] Hu J, Wang L, Li F, Han YL, Lin M, Lu TJ, et al. Oligonucleotide-linked gold nanoparticle aggregates for enhanced sensitivity in lateral flow assays. Lab Chip. 2013; 13: 4352-7.

[S2] Gomez-Martinez J, Silvy M, Chiaroni J, Fournier-Wirth C, Roubinet F, BaillyP, et al. Multiplex Lateral Flow Assay for Rapid Visual Blood Group Genotyping. Anal Chem. 2018; 90: 7502-9.

[S3] Qiu W, Xu H, Takalkar S, Gurung AS, Liu B, Zheng Y, et al. Carbon nanotube-based lateral flow biosensor for sensitive and rapid detection of DNA sequence. Biosens Bioelectron. 2015; 64: 367-72.

[S4] Yang M, Zhao Y, Wang L, Paulsen M, Simpson CD, Liu F, et al. Simultaneous detection of dual biomarkers from humans exposed to organophosphorus pesticides by combination of immunochromatographic test strip and ellman assay. Biosens Bioelectron. 2018; 104: 39-44.

[S5] Liu X, Zhang C, Liu K, Wang H, Lu C, Li H, et al. Multiple SNPs Detection Based on Lateral Flow Assay for Phenylketonuria Diagnostic. Anal Chem. 2018; 90: 3430-6.
